# *In vivo*-like 3-D model for sodium nitrite- and acrylamide-induced hepatotoxicity tests utilizing HepG2 cells entrapped in micro-hollow fibers

**DOI:** 10.1038/s41598-017-13147-z

**Published:** 2017-11-01

**Authors:** Qiang Chu, Yiying Zhao, Xuer Shi, Wen Han, Yanzhen Zhang, Xiaodong Zheng, Jing Zhu

**Affiliations:** 1 College of Biosystems Engineering and Food Science, Zhejiang University, No. 866 Yuhangtang Road, Hangzhou, 310058 P.R. China; 2 Department of General Dentistry, The Second Affiliated Hospital of Zhejiang University School of Medicine, No. 88 Jiefang Road, Hangzhou, 310009 P.R. China; 3 Department of stomatology, Hangzhou First People’s Hospital, Nanjing University, No. 261 huansha Road, Hangzhou, 310006 P.R. China

## Abstract

To address the need for a high throughput toxicity test in the modern food industry, an *in vivo*-like 3-D cell model was constructed in this study to provide an alternative to controversial long-term animal models and to improve the sensitivity and accuracy of the traditional monolayer model. The model formed cell cylindroids within polyvinylidene fluoride (PVDF) hollow fibers and therefore mimicked the microenvironment of liver tissue. Microscopy methods were used, and liver-specific functions were measured to demonstrate the superiority of the model compared to the monolayer model, as well as to optimize the model for best cell performances. Later, toxicity tests of sodium nitrite and acrylamide were conducted in both the 3-D model and the monolayer model to study the sensitivity of the 3-D model in toxicity responses. As expected, HepG2 cells within the 3-D model responded at lower concentrations and shorter exposure times compared to cells within the monolayer model. Furthermore, western blot analysis of apoptosis pathways also supported the argument.

## Introduction

Due to safety concerns, nutritional enhancement and pure convenience, the production and consumption of processed foods among the general public have increased worldwide, playing an irreplaceable role in daily dietary patterns. However, with an increasingly health-conscious population, concerns over processed foods have intensified, and a number of them have been well-confirmed^1,2^. Currently, in a market of massive processed food products, accurate and efficient evaluations of either the various food additives or the chemicals produced through the manufacturing process are in great demand and are urgently required.

Typical safety evaluation methods, including toxicity tests of food components, are usually conducted using animal models. Though widely accepted and used, animal models fail to perform accurate predictions of the toxic effects due to differences in species-dependent hepatic metabolisms and different sensitivities to chemicals^3^ along with high costs and long experimenting periods^4^. However, by culturing cells*, in vitro* models provide an option to overcome these limitations and help construct a more tissue-like environment. A traditional monolayer culture^5^ is the most widely used 2-D culturing method that manages to maintain high bioactivity and proliferation ability in a relatively short time, which therefore supports high throughput screening. However, after several days of culturing, the monolayer cells lose partial differentiation and secretion abilities^6^, activities of phase I and II metabolic enzymes^7,8^ along with significant decreases in urea and albumin synthesis^9^. The flattened morphology of the cells, as well as the rapid disappearance of bioactivities, means the model deviates from a real *in vivo* 3-D microenvironment^10,11^.

To address the limitations of 2-D models, various 3-D culture systems^12–14^ have been developed to form hepatocyte organoids, leading to better long-term cell viability and bioactivity. Liver cell organoids, including spheroids and cylindroids, can maintain high-level liver functions *in vitro* due to the presence of abundant cytoplasmic organelles and close cell-cell contacts^15–17^. Therefore, hepatocyte organoid cultures have been said to best mimic the *in vivo* microenvironment of liver tissues, and this type of culture has been used to study the development of human organs using stem cells^18^. In addition, among the hepatocyte organoids, cylindroids showed better performances over liver-specific functions than spheroids^19^ and can form a larger tissue-like structure because of the cylindrical structures inside the model^14^. Our approach is based on bioreactor technology^20^ and using biomaterials that can generally provide structural support and strengthen cell-cell interactions to mimic the *in vivo* microenviroment^21^. Specifically, we used biocompatible hollow fibers as cellular scaffolds to form hepatocyte cylindroids^22^.

In regard to cell line usage, the HepG2 cell line is one of the most widely used models for toxicity tests for chemicals^23,24^ and has better sensitivity compared to other cell lines^25^. Though primary hepatocytes are preferred for biotransformation studies due to the resemblance to an *in vivo* environment, the high expression of phase II genes makes the HepG2 cell line a useful model to study drug metabolisms^26^. Moreover, to form an affordable, high throughput model, the HepG2 cell line is well reputed for its easiness to handle and can maintain a reproducible human system, while primary hepatocytes are rarely available and can lose metabolic activity over the long term

To study the efficiency and sensitivity of the model in food toxicity tests, two common chemicals in processed foods were selected as sample compounds for primary application. Acrylamides (AAs) have been found in various processed foods, with the highest levels being observed in fried potato products, bread, and bakery wares^27^. The major mechanism for acrylamide formation in foods is the Millard reaction with asparagine and carbonyl-containing compounds as precursors^28^ through frying, baking, and roasting processes. Acrylamide’s neurotoxicity has been well-documented in human epidemiological studies, while reproductive toxicity, genotoxicity, clastogenicity and carcinogenicity remain as potential human health risks according to animal studies only^29^. Sodium nitrite is widely used as a food additive in cured meat to preserve the quality, appearance, and flavor of the products^30^. Nitrite itself is said to be 10 times more toxic than nitrate, and high levels of nitrites have caused food-related deaths in Germany^31^. Additionally, nitrite is responsible for carcinogenic nitrosamine formation when reacting with secondary amines under heat and acidic conditions^31^. In 2015, IARC declared processed meats to be a Group 1 carcinogen according to data related to colorectal cancer and stomach cancer, while red meat consumption was classified as probably carcinogenic to humans^32^. Both the benefits and possible harmful effects of these well-known chemicals have aroused great interest and debates over the proper intake amount of food additives over years until now. Though regulations and animal-based studies have been performed, more accurate *in vivo* studies have not been conducted based on ethical reasons, and the current testing models struggle to set accurate intake limits for all types of foods due to their great varieties and fast product renewal.

The objective of this study was to build an optimized model to conduct hepatotoxicity tests on compounds in processed foods and thus improve the efficiency, sensitivity, and accuracy of traditional safety evaluation models. To this end, the model was first constructed using PVDF hollow fibers as the supportive scaffolds and collagens for better cell adhesion and integrity within the model^20^. Bioactivity parameters, such as albumin and urea secretion, were measured later to set the optimum culture conditions for the model. To evaluate the superiority of the 3-D model compared with the traditional monolayer models, differences in cell viability and other bioactivity performances were examined to confirm the assumption. Finally, the primary application of the model in food areas was conducted by examining the hepatotoxicity of AA and nitrite. Hopefully, this model could be used further in high throughput screening tests to set upper intake limits for processed food compounds and study the possible harmful effects in an *in vivo-*like microenvironment.

## Results and Discussion

### Characterization of the PVDF hollow fiber materials

**Figure 1:**
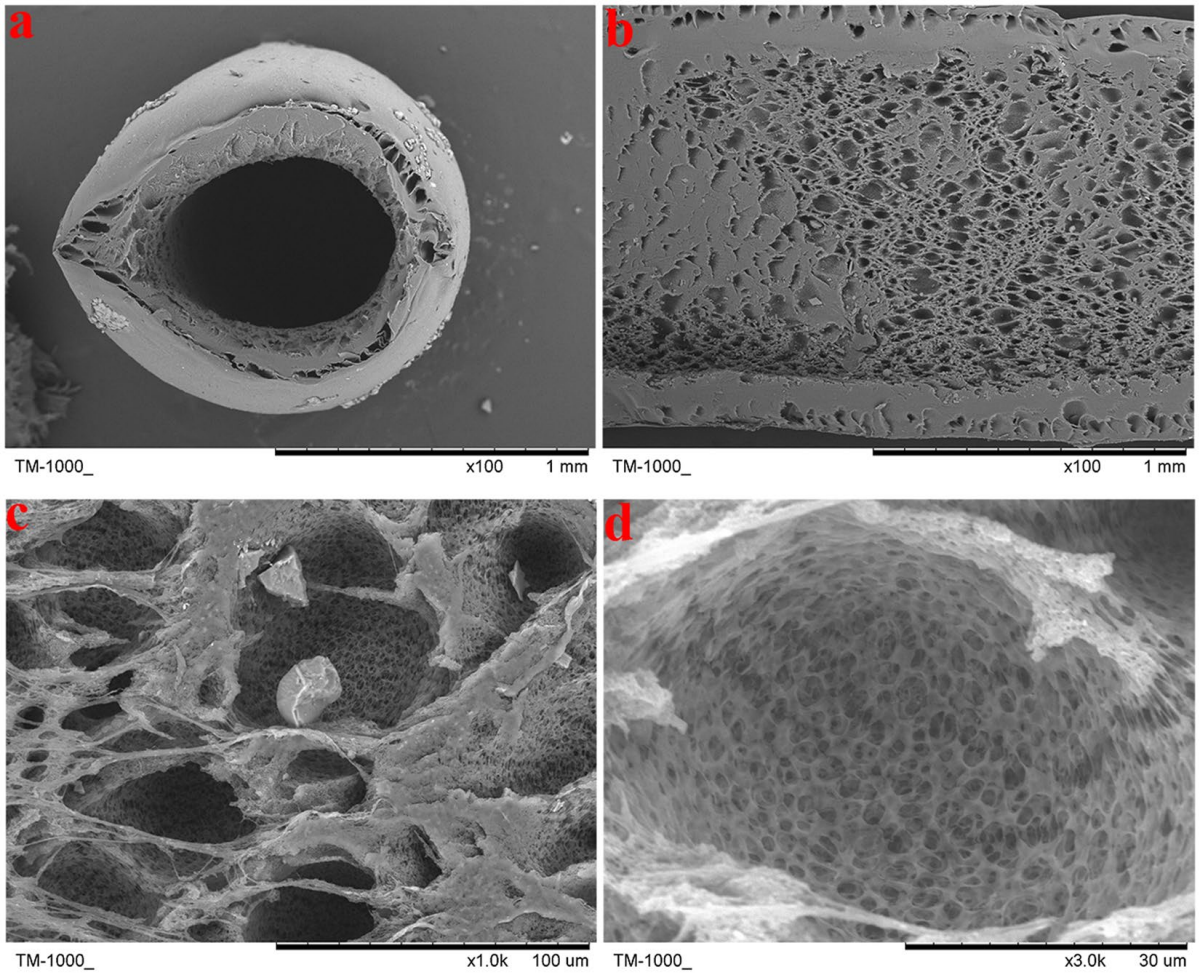
SEM images of PVDF hollow fibers after sterilization. (**a**) is the 100× cross cut image of hollow fibers after sterilization. (**b,c,d**) are the straight cut images of hollow fibers after sterilization with amplification factors of 100×, 1000×, and 3000×, respectively.

Though known to have great performance in chemical stability, radiation resistance, heat resistance^33^ and other general properties, a more thorough look at the material was needed to assure its compatibility with cell growth. Therefore, SEM photographs of the polyvinylidene fluoride (PVDF) hollow fiber were made from different directions (cross cut and straight cut) and using different amplification factors. The photographs are shown in Fig. 1.

As expected, the inner surface of the material is rough and thus favors adherence, growth, proliferation and functional expressions of the cells^34^. Additionally, the massive micropores meet the demand of exchanging materials between the inner cells and the outside media. All of these characteristics showed the feasibility of using sterilized PVDF to construct a bioreactor due to its high permeability and capacity for maintaining cell growth^35^.

### Construction and optimization of the 3-D model

**Figure 2:**
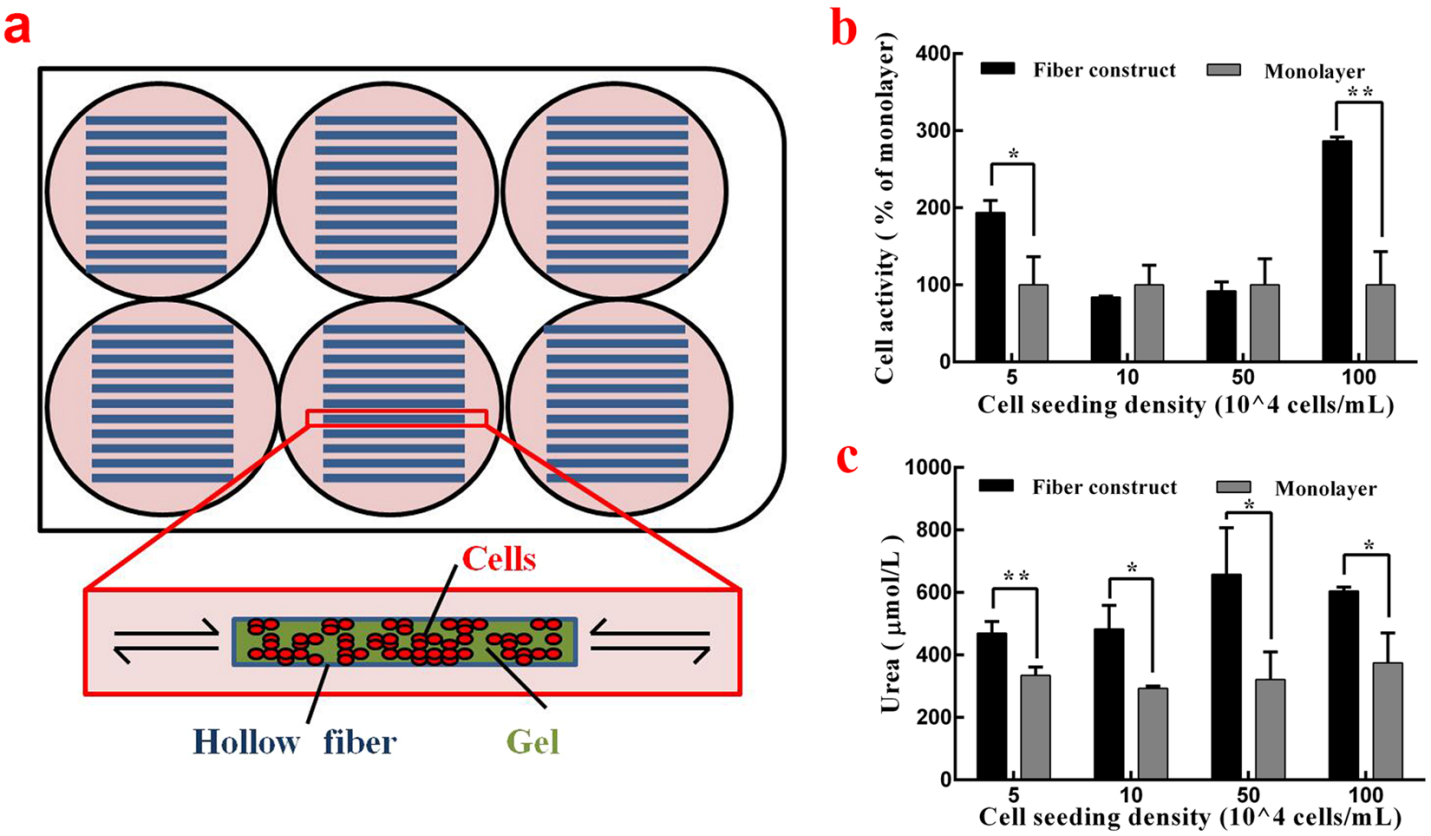
(**a**) Schematic diagram of the 3-D model. (**b**). (**c**) The influence of different cell seeding densities on cell activity performance in the hollow fiber model and monolayer model with graph b showing cell viability and graph c showing urea secretion.

Figure 2a illustrates the *in vivo*-like 3-D model. PVDF hollow fibers with massive micropores were used as scaffolds, assuring an efficient material exchange between the inner hepatocytes and the culture medium while providing sufficient space and protection for cell growth. Mixed with collagen solutions, they have two major functions here: to enable the gathering of cells so cylindroids can be formed within the hollow fibers and to improve the long-term bioactivity and integrity of the cells through better adhesion to the hollow fiber^20^, for which cell suspensions were injected into the hollow fibers. The fibers were placed within a petri dish, and no fluid was pumped through the inner channels. Taken together, all of these materials helped to build up a more tissue-like *in vitro* microenvironment for further study.

After the primary construction of the model, the cell seeding density was optimized to assure the best performance of the cell cylindroids. Mixed with a 2 mg/mL collagen solution, a cell suspension of different cell concentrations from 5 × 10^4^ to 1 × 10^6^ cells/mL were injected into the hollow fiber model and the petri dish. The number of hollow fibers placed in one petri dish was calculated to assure an equal cell growth area in the 3-D model and the monolayer model. After 2 days of cultivation, the cell viability of the two models was determined using an MTT method and the performance in the monolayer model was set at 100%. As seen from Fig. 2b, cell viability in the 3-D model was significantly greater than the monolayer model at a cell seeding density of 5 × 10^4^ cells/mL and 1 × 10^6^ cells/mL. At a cell seeding density of 1 × 10^5^ cells/mL and 5 × 10^5^ cells/mL, a slightly higher viability in the monolayer model was observed. Urea synthesis was also measured under different seeding densities, and the result is shown in Fig. 2c. In general, urea secretion in the 3-D model remained greater at all seeding densities, and the difference between the two models became more significant at the higher density. The limited growing space of the petri dish may well explain the result, since the urea secretion in the monolayer model showed a slight increase as the density was multiplied. Therefore, the superiority of the 3-D model was reconfirmed; that is, it provided enough space to create a microenvironment for cell growth. Considering the results showed in Fig. 2b and c, 1 × 10^6^ cells/mL was selected as the optimal cell seeding density when both the cell viability and urea synthesis remained at a high level.

### General morphology and structure of the 3-D model

**Figure 3:**
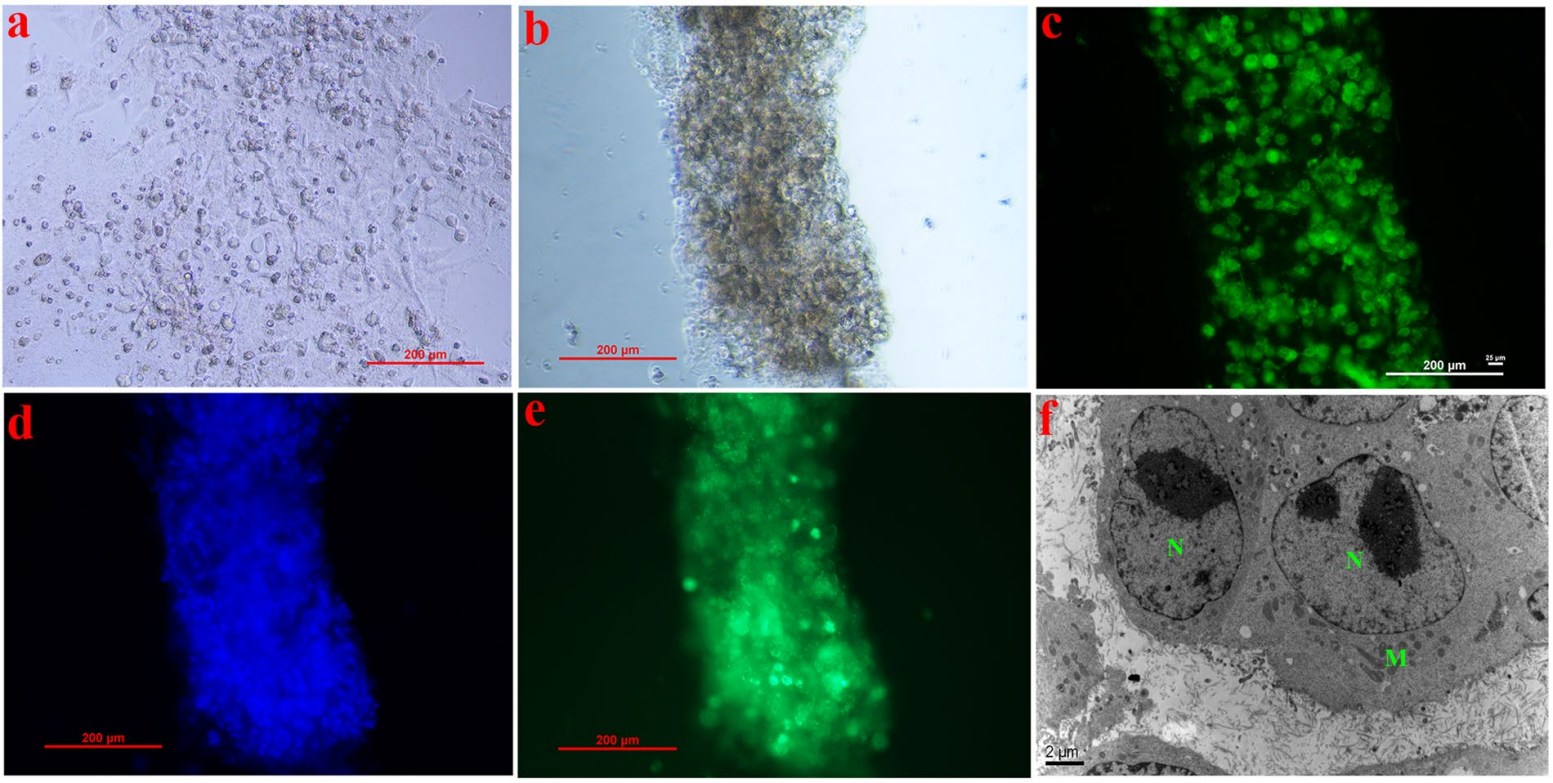
(**a**) Light image of the cell aggregates without out hollow fibers after 48 h culture (**b**) light image of the cylindroid after 48 h culture, (**c**) mitochondria membrane potential (MMP) image of the cylindroid after 48 h culture, (**d**) dyed nuclei image of the cylindroid after 48 h culture, (**e**) phalloidine stain of the cylindroid after 48 h culture, (**f**) transmission electron microscope (TEM) image of the cylindroid after 48 h culture where N stands for nuclei, M stands for mitochondria.

To illustrate the indispensable function of hollow fibers as scaffolds, the morphology of cell aggregates without the material was studied under a microscope after 48 h culture. As seen from Fig. 3a, as with the traditional monolayer model, cells formed a flattened and scattered 2-D layer that floated in the culture media. As shown in Fig. 3b, a well-organized 3-D cell cylindroid was clearly observed. Corresponding to Fig. 3b,c and d illustrated the overall morphology of the cylindroids, showing a compact and well-organized network of cell aggregates in a favorable growth situation with great performances of mitochondrial activity and well-dyed nuclei.

The overall structure of the cell cylindroid was studied with the help of fluorescent phalloidin, as shown in Fig. 3e. Combined with F-actin, the uniform fluorescence intensity indicated a well-organized cytoskeleton formed within the cell aggregates that highly mimicked the *in vivo* environment^36^ and maintained cellular morphology. For the microstructure, the TEM image in Fig. 3f revealed a clear morphological appearance of individual cells inside the cylindroids where smooth and integrate nuclei (N) were found inside the cells along with abundant mitochondria (M), corresponding to the previous Fig. 3c and d.

The above images of structures and cell activity performances demonstrated well-organized and compact cell aggregates with extracellular matrix entrapment, strongly suggesting the success of the model in forming *in vivo*-like hepatocytes cylindroids. Thus, this method of cell culture’s mimicking the *in vivo* microenvironment was better than the traditional monolayer culturing method.

### General evaluation of the 3-D model

**Figure 4:**
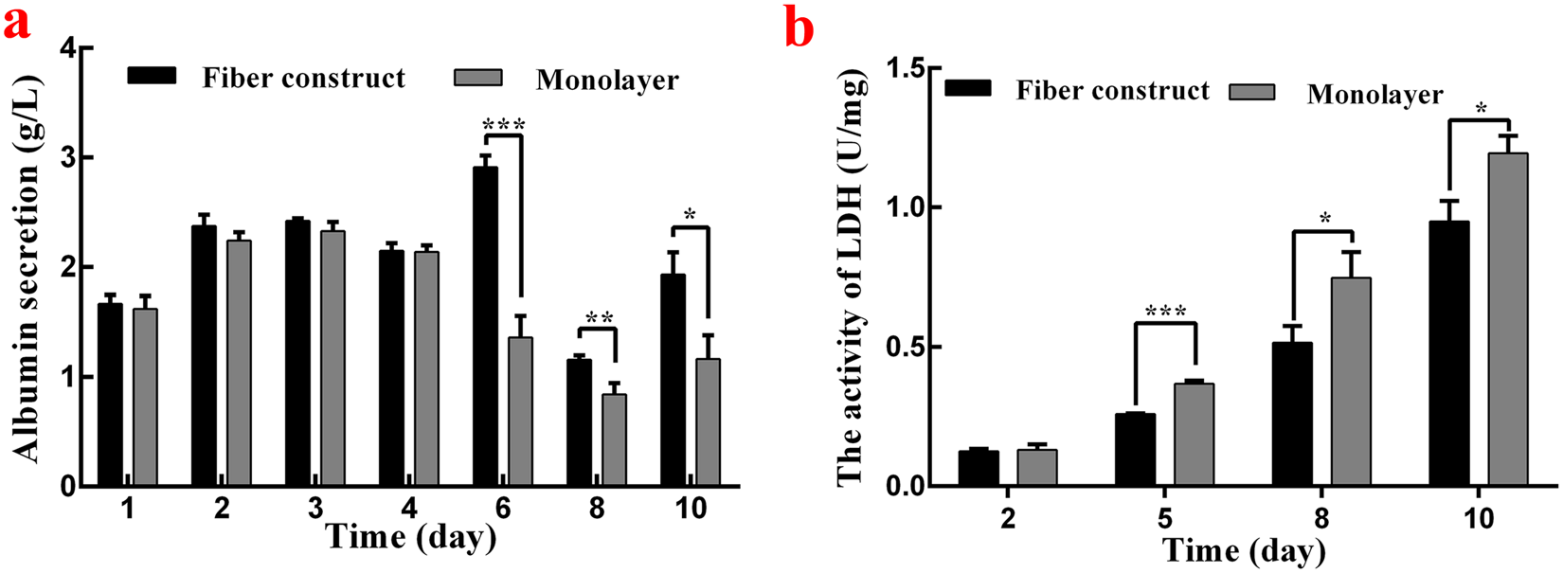
(**a**) Time-related albumin secretion in a 3-D model and monolayer model b time-related lactate dehydrogenase (LDH, **b**) in 3-D model and monolayer model.

The metabolic activities of HepG2 cells in both the 3-D model and monolayer model were monitored through the entire cultivation period for 10 days, and the cell density was set at 1 × 10^6^ cells/mL in both models in the following comparisons. Albumin secretion and LDH leakage were measured using the cell culture medium, indicating the superiority of the overall metabolic performances of cells within the 3-D model (Fig. 4).

During the cultivation period, phase contrast microscopy was used to observe the morphological change of monolayer cells from massive cell adherence and proliferation to gradual detachment and lysis. The observation results are well demonstrated in Fig. 4a. As expected, albumin secretion of both models increased significantly during the first 3 days, suggesting a strong growth ability, synthetic ability and secretory ability of the cells in the earlier cultivation period. However, in the following 6 days, albumin secretion of the monolayer model decreased accordingly, while the 3-D model had better performance compared with the monolayer model and showed the greatest advantage on day six. For the 3-D model, the decrease in albumin secretion at day 8 could be explained by cell apoptosis, yet the larger specific surface area benefited cell attachment and thus supported the growth of cell aggregates, leading to a recovery of albumin secretion at day 10.

Therefore, one of the superiorities of the 3-D model lies in its ability to maintain cell growth and good performance over a relatively long period, and the result is shown in Fig. 4b. Along with increasing the cultivation time, the LDH leakage increased in both models with a generally greater amount in the monolayer model, indicating higher potential cell damage compared with the 3-D model. Unsurprisingly, the difference between the two models grew larger as time went by and thus confirmed the advantage of the 3-D model in long-term maintenance of a high-level cell activity. Considering the two figures combined, along with the *in vivo*-like microenvironment concluded from microscopy observations, the superiority of using the 3-D model to conduct long-term toxicity studies *in vitro* was underscored.

### Primary model application using sodium nitrite and acrylamide as samples

**Figure 5:**
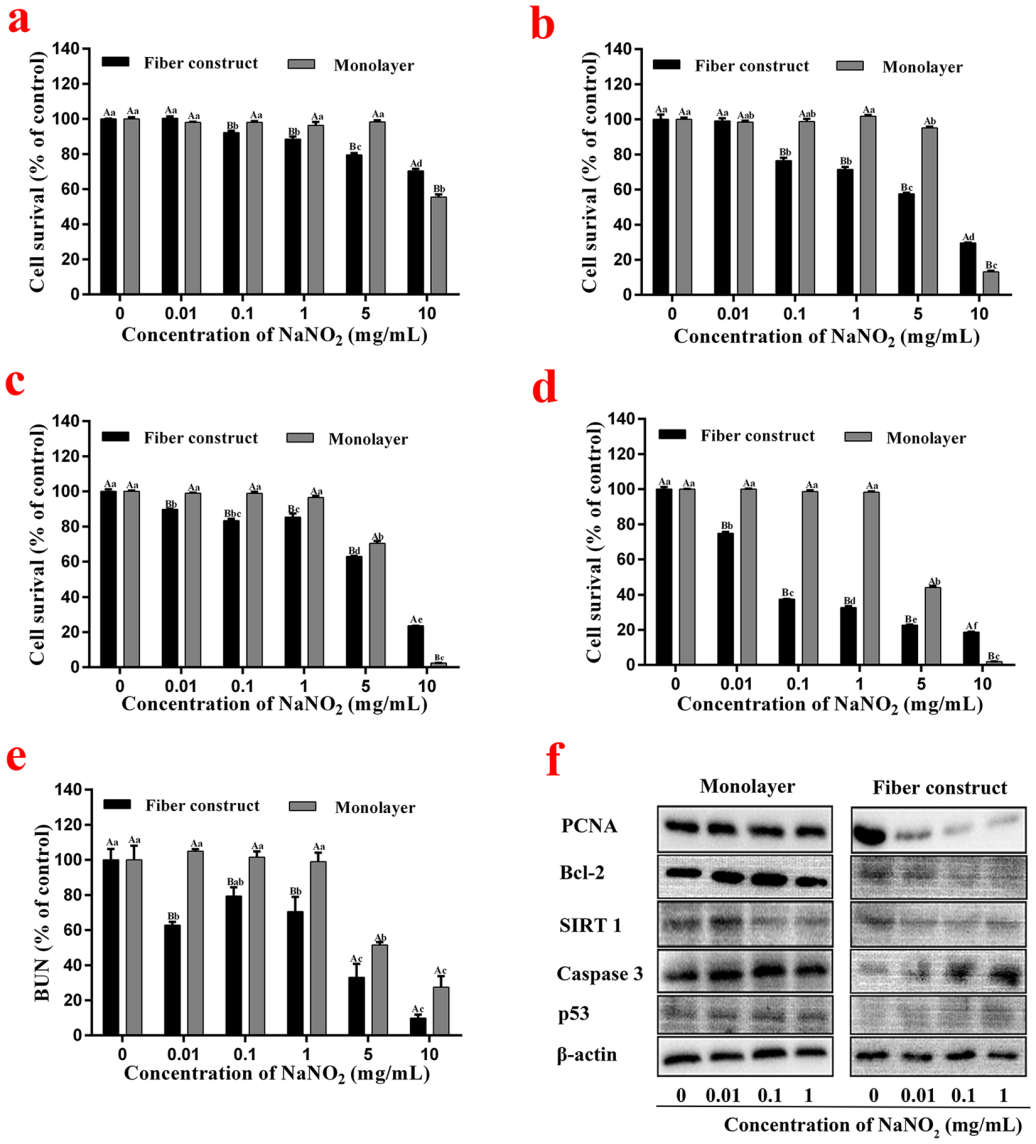
(**a–e**) Cell viability and metabolic performance after different exposure times (a:12 h b:24 h c:48 h d:72 h e:24 h, urea secretion) to different concentrations of sodium nitrite (0 mg/ml as the control group) in both models. (**f**) Western-blotting analysis of key apoptosis proteins in both models after 48 h culture.

Sodium nitrite was selected as the first sample compound to be tested in the model. The cell viability of the HepG2 cells exposed to different concentrations of the compound within the two models was monitored throughout the entire cultivation period. After a certain time (12 h, 24 h, 48 h, or 72 h), the cell viability of the cells that were not exposed to sodium nitrite (0 mg/mL exposure) was set as 100% for further comparisons of the models. The results are shown in Fig. 5a–d.

After 12 h and 24 h exposures, the cell viability of cells within the 3-D model gradually decreased as concentrations increased from 0.01 mg/mL to 10 mg/mL, reaching a significant difference with the control group (0 mg/mL) at 0.1 mg/mL, while the monolayer cells only experienced a significant decline until 10 mg/ml. The same tendency is shown in Fig. 5c and d where 3-D model cells responded at 0.01 mg/mL and monolayer cells at 5 mg/mL after 48 h and 72 h exposures. The influence of the NaNO_2_ application was also confirmed regarding urea synthesis after 24 h exposure (Fig. 5e), which demonstrated the adverse effects of the compound with a rapid dose-dependent decrease in the 3-D model compared with a slow change in the monolayer model. To further understand the mechanisms causing the observed differences in toxicity responses, we performed western-blot analysis for several key apoptosis proteins^37–39^ after 48 h of culture in both models. As seen from Fig. 5f, gene expressions remained the same in the monolayer model regardless of the compound concentration. However, in the fiber constructed model, expressions of PCNA, Bcl-2, and SIRT1 decreased as the concentration of sodium nitrite increased, accelerating cell apoptosis, while the up-regulated expression of Caspase3 and p53 led to the same result.

**Figure 6:**
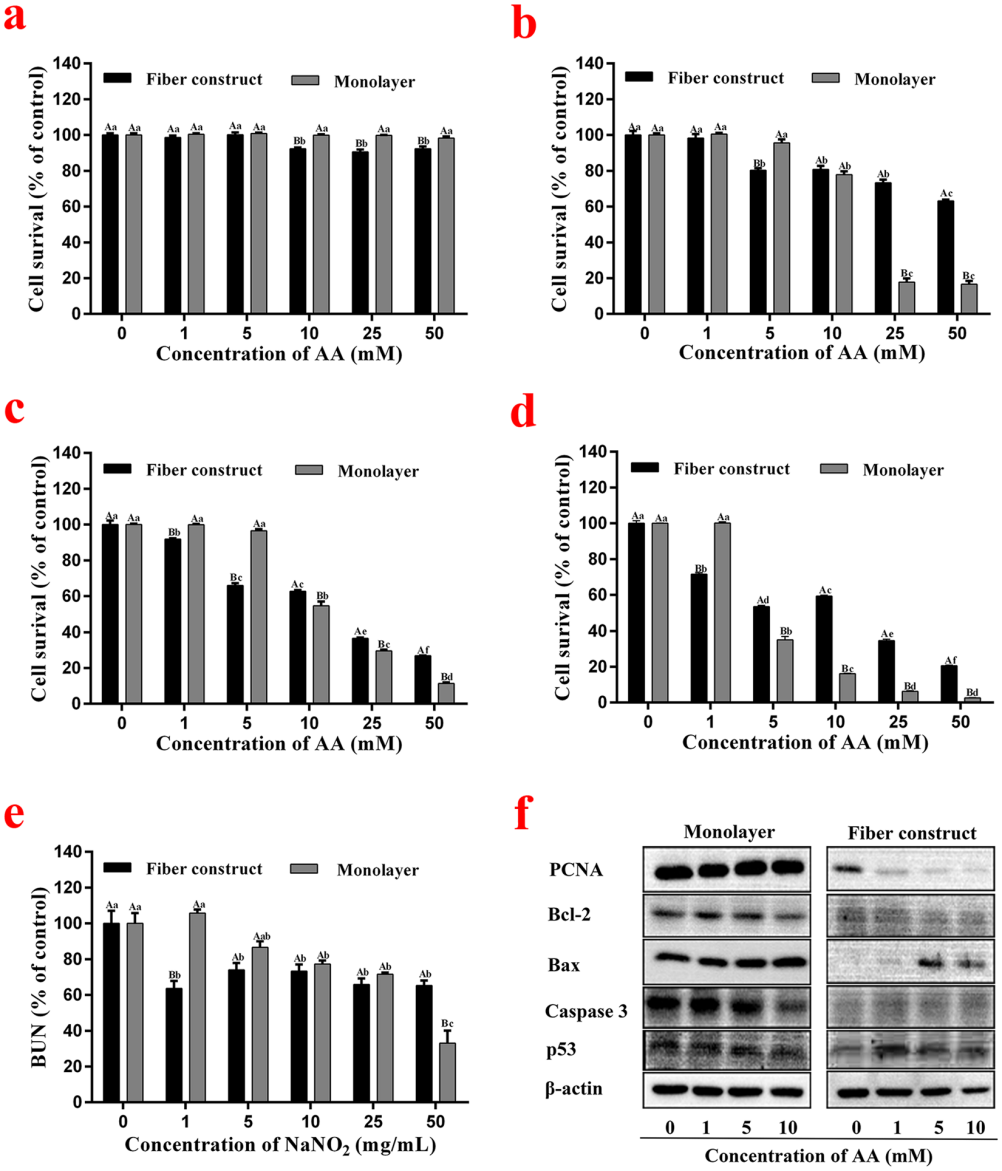
(**a–e)** Cell viability and metabolic performance after different exposure times (a:12 h b:24 h c:48 h d:72 h e:24 h, urea secretion) to different concentrations of acrylamide (0 mM as the control group) in both models. (**f**) Western-blotting analysis of key apoptosis proteins in both models after 48 h culture.

Both the 3-D model and the monolayer model were later treated with acrylamide at different concentrations to reconfirm the tendency presented in the previous application with sodium nitrite. Figure 6a–d shows the results of an MTT analysis in the form of cell viability where the values of the control group were set as 100%. After exposure for 12 h, 3-D model cells showed a dose-dependent response starting from 10 mM, while the monolayer cells remained unchanged. As shown in Fig. 6b, the responsive concentration of the 3-D model and monolayer model were 5 mM and 10 mM, respectively. Figure 6c and d show both of the downward trends in the 3-D model started at 1 mM, while the decline appeared later in the monolayer model. Interestingly, cell viability in the 3-D model remained greater at a higher concentration compared with the monolayer model, especially where the acrylamide treatment lasted for 72 h (Fig. 6d). The *in vivo*-like environment of the 3-D model may explain the lower death rate, since well-organized liver cells in the body perform efficient detoxification mechanisms, which help compromise the long term toxicity of the compound^40^. In addition, after a certain time of exposure to acrylamide, a significant decrease in urea synthesis of HepG2 cells within the 3-D model was observed, respectively, indicating the poorer metabolic activities of cells due to acrylamide toxicity (Fig. 6e). As Fig. 5f and Fig. 6f show, the same trends that correspond with the apoptosis pathways were observed, where the gene expressions changed to as low as 1 mM in the fiber-constructed model and remained the same in the monolayer model.

In general, cell viability data in line with functional readouts clearly showed the sensitivity of the 3-D model in toxicity tests compared with the traditional monolayer model. In both applications, the 3-D model cells started to present responses after 12 h exposure, while the monolayer cells remained unaffected. Additionally, the responsive concentration appeared to be many times lower in the 3-D model regardless of treatment time or compound types. Further functional measurements of urea synthesis also verified the sensitivity of the 3-D model when clear does-dependent trends appeared at low concentrations. Moreover, western blot analysis over apoptosis pathways reconfirmed the previous observations where the monolayer model hardly responded compared to the rather sensitive fiber construct model. Conclusively, cells in the 3-D model were influenced more by the sample compounds than the monolayer cells.

In the real *in vivo* test, the plasma concentration of sodium nitrite and its metabolites was approximately 140 $\mu$mol/L when clinical changes were observed in healthy human objects^41^. In our experiment, the fiber constructed model responded at 144.9 $\mu$mol/L while the monolayer model responded at 1449 $\mu$mol/L. For the *in vivo* test of acrylamide, moderate levels of behavioral neurotoxicity were observed in rats when plasma concentrations of AA reached 30.1 $\mu$g/mL after repeated i.p injections^42^. Accordingly, in our study, the fiber constructed model responded at 71.08 $\mu$g/mL while the monolayer model responded at 710.8 $\mu$g/mL. Though differences remain between the reference data and the experimental data due to the disparity in study contexts, experimental data of both drugs in the 3-D model still approached the toxic concentration *in vivo* compared with the monolayer model, further demonstrating the superiority of the designed model.

## Materials and Methods

### Materials

PVDF (Polyvinylidene Fluoride) hollow fiber materials (aperture 0.2 μm, pure water flux 800 L/m^2^h under 0.1 Mpa, inner/outer diameter 0.7/1.3 mm) were purchased from SENUO Filtration Technology Co., Ltd. (Tianjin, China). Rat-tail collagen, MTT, Hoechst 33258 dyes were purchased from Sigma-Aldrich (St. Louis, MO, USA). Phalloidine dye was purchased from Yeasen Biotechnology Institute (Shanghai, Chiana). Urea, albumin and LDH kits were purchased from Nanjing Jiancheng Bioengineering Institute (Nanjing, China). Fetal Bovine Serum (FBS) and cell lysis buffer were purchased from Beyotime Institute of Biotechnology, Ltd. (Shanghai, China). Sodium nitrite and acrylamide were purchased from Aladdin Reagent (Shanghai, China). All chemical reagents used were of analytical grade.

### Cell culture and treatments

For the monolayer culture, HepG2 cells were cultured in DMEM medium (Gibco) containing 100 units/mL of streptomycin, 100 units/mL of penicillin and 10% of FBS and was later incubated in 5% CO_2_ at 37 °C.

For the 3-D culture, DMEM culture medium was first mixed with 2 g/L collagen at a ratio of 1:1 for later usage. HepG2 cells were counted for the appropriate seeding density and were then mixed with the previous solution. After adequate mixing, 20 μL of the final solution was injected into the lumen of a 3 cm-long sterilized PVDF hollow fiber. To stabilize the collagen, the hollow fibers were kept for 10 min at 37 °C. Later, a certain number of hollow fibers were put into the 6-well plate with 2 mL preheated culture medium and were incubated in 5% CO_2_ at 37 °C. After reaching the appropriate confluence, the cells were washed with PBS twice and treated with different concentrations of sodium nitrite and acrylamide diluted in the culture medium. Cells with no treatment (medium only) were used as the negative control.

### Cell viability assay

For the monolayer culture, cells were seeded into a 96-well plate at a density of 5 × 10^3^ cells per well for 24 h and then treated with sodium nitrite and acrylamide at the indicated concentration for 12 h, 24 h, 48 h or 72 h. Next, HepG2 cells were immersed within 0.5 mg/mL MTT for 4 h in the incubator in 5% CO_2_ at 37 °C. At the end of the incubation period, 150 μL DMSO was added to dissolve the formazan precipitate. The absorbance was measured at 570 nm using a spectrophotometer.

For the 3-D culture, the hollow fiber reactors were immersed within 2 mL of 0.5 mg/mL MTT-phosphate buffer solution in each well of the 6-well plate, which was later incubated for 4 h. At the end of the incubation, the hollow fibers were transferred into a 2 mL centrifuge tube and the generated formazan precipitate was dissolved using 750 μL DMSO. The absorbance was measured using a spectrophotometer at the wavelength of 570 nm.

### Phalloidine dying

The hollow fibers were first taken out and cut in the straight direction. The cells were scraped from the inner surface of the hollow fibers and were then immersed within Phalloidine dye solution for 30 min. Later, the dye solutions were discarded and PBS was used to rinse. The cells were observed under the phase contrast fluorescence microscope.

### Fluorescent staining of nuclei

The hollow fibers were first taken out and cut in the straight direction. The cells were scraped from the inner surface of the hollow fibers and were then immersed within 10 μg/mL Hoechst33258 solutions for 30 min cultivation at 37 °C. Later, the dye solutions were discarded and PBS was used to rinse. The cells were observed under the phase contrast fluorescence microscope.

### MMP image

The hollow fibers were first taken out and cut in the straight direction. The cells were scraped from the inner surface of the hollow fibers and were then immersed within Rhodamine 123 dye solution for 30 min cultivation at 37 °C. Later, the dye solutions were discarded and PBS was used to rinse. The cells were observed under the phase contrast fluorescence microscope.

### Assays on liver-specific functions

After days of cultivation, the culture media were sampled and assays including urea synthesis, albumin secretion, and LDH leakage were performed using kits purchased from Nanjing Jiancheng Bioengineering Institute (Nanjing, China).

### Western blot analysis

Equal amounts of proteins were separated with 10% SDS-PAGE and transferred to a low fluorescence PVDF membrane (Millipore, Billerica, MA). The membrane was blocked with 5% (w/v) nonfat dry milk in TBS buffer containing 0.1% Tween 20 (TBST; 25 mM Tris/HCl, 140 mM NaCl, 0.1% Tween 20, pH 7.5) for 1 h. The membrane was incubated with primary antibodies (Abcam, Shanghai, China) and horseradish peroxidase-conjugated secondary antibodies. Proteins were detected with enhanced chemiluminescent reagents (Beyotime, Shanghai, China).

### Result analysis

All experiments were carried out at least three times. The results were expressed as the mean ± standard deviations (SD) and analyzed with a one-way ANOVA using SPSS (version19.0). P < 0.05 (* = 0.05, ** = 0.01, *** = 0.005) was considered to be significant, and graphs were drawn with GraphPad Prism 6.0 for Windows (GraphPad Software, San Diego, CA, USA).

## Conclusions

Overall, the *in vivo*-like 3-D HepG2 model was successfully constructed through PVDF hollow fiber materials and a fiber-constructed culture. The morphology of the cell cylindroids formed within the bioreactor was illustrated under microscopy observation, while the superiority of the model in cell metabolic activities and long-term maintenance of liver-specific functions were further evaluated before application. Later, toxicity tests of sodium nitrite and acrylamide were conducted in both models in the form of MTT assays after certain exposure times. Meanwhile, metabolic performances were also measured to study the possible mechanism of the toxicity induced by the chemicals. General results suggested toxicity for both chemicals, leading to cell death and a decrease in urea synthesis. Additionally, a comparison between the two models revealed the sensitivity of the 3-D model with a clear response to chemicals at a lower dose and shorter exposure time. Therefore, this study provides potential solutions for the increasing demand for accurate and efficient high throughput toxicity tests for food additives and food-borne compounds through mimicking *in vivo*-like microenvironments.

## Electronic supplementary material

Supplementary Inforamtion
