# Supplementary material for: In vivo-like 3-D model for sodium nitrite- and acrylamide-induced hepatotoxicity tests utilizing HepG2 cells entrapped in micro-hollow fibers
Source: Sci Rep. 2017 Nov 1;7:14837. doi: 10.1038/s41598-017-13147-z (PMC5665964; doi:10.1038/s41598-017-13147-z)
Supplement: Supplementary file 1 — Supplementary Inforamtion [file 41598_2017_13147_MOESM1_ESM.pdf]

In vivo-like 3-D model for sodium nitrite- and acrylamide-induced hepatotoxicity tests utilizing HepG2 cells entrapped in micro-hollow fibers

Qiang Chu[1], Yiying Zhao[1], Xuer Shi[1], Wen Han[1], Yanzhen Zhang[2], Xiaodong Zheng[1*], Jing Zhu[3*]

[1] College of Biosystems Engineering and Food Science, Zhejiang University, No.866 Yuhangtang Road, Hangzhou, 310058, P.R. China

[2] Department of General Dentistry, The Second Affiliated Hospital of Zhejiang University School of Medicine, No.88 Jiefang Road, Hangzhou, 310009, P.R. China

[3] Department of stomatology, Hangzhou First People's Hospital, Nanjing University, No.261 huansha Road, Hangzhou, 310006, P.R. China

*Corresponding author: xdzheng@zju.edu.cn

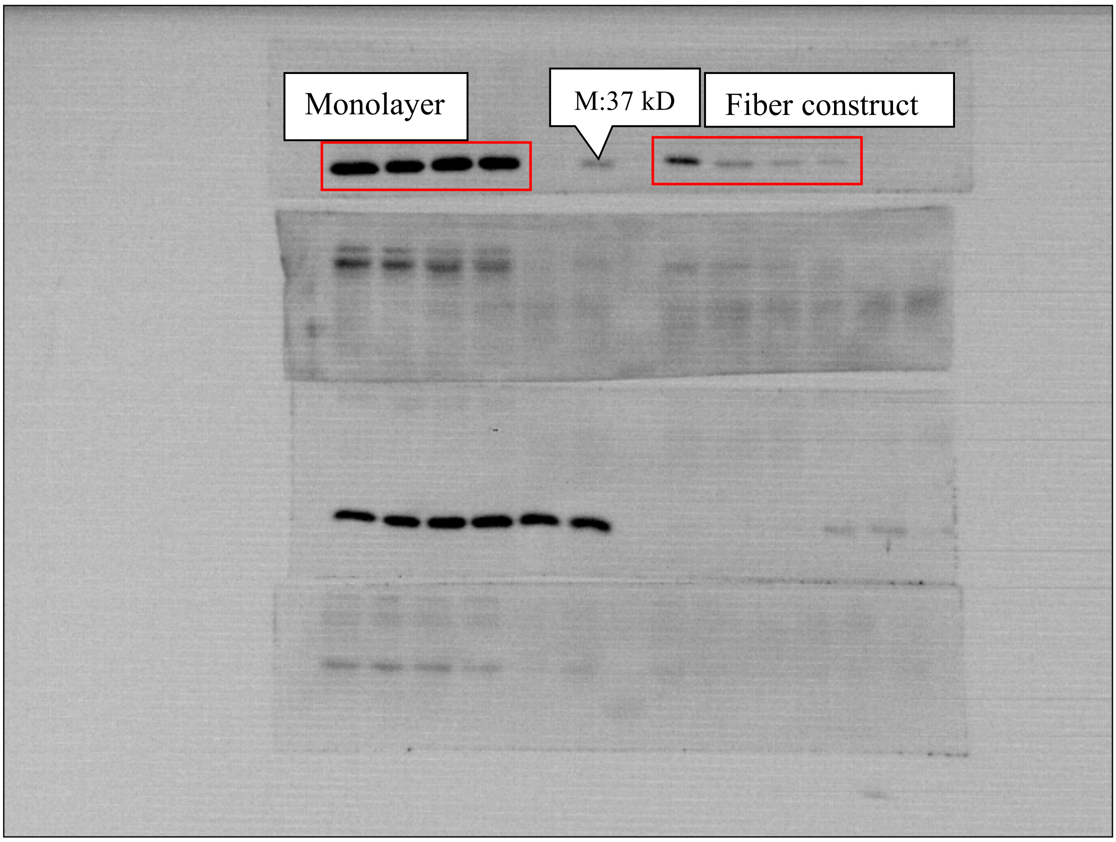

Figure 1.1 Acrylamide-PCNA

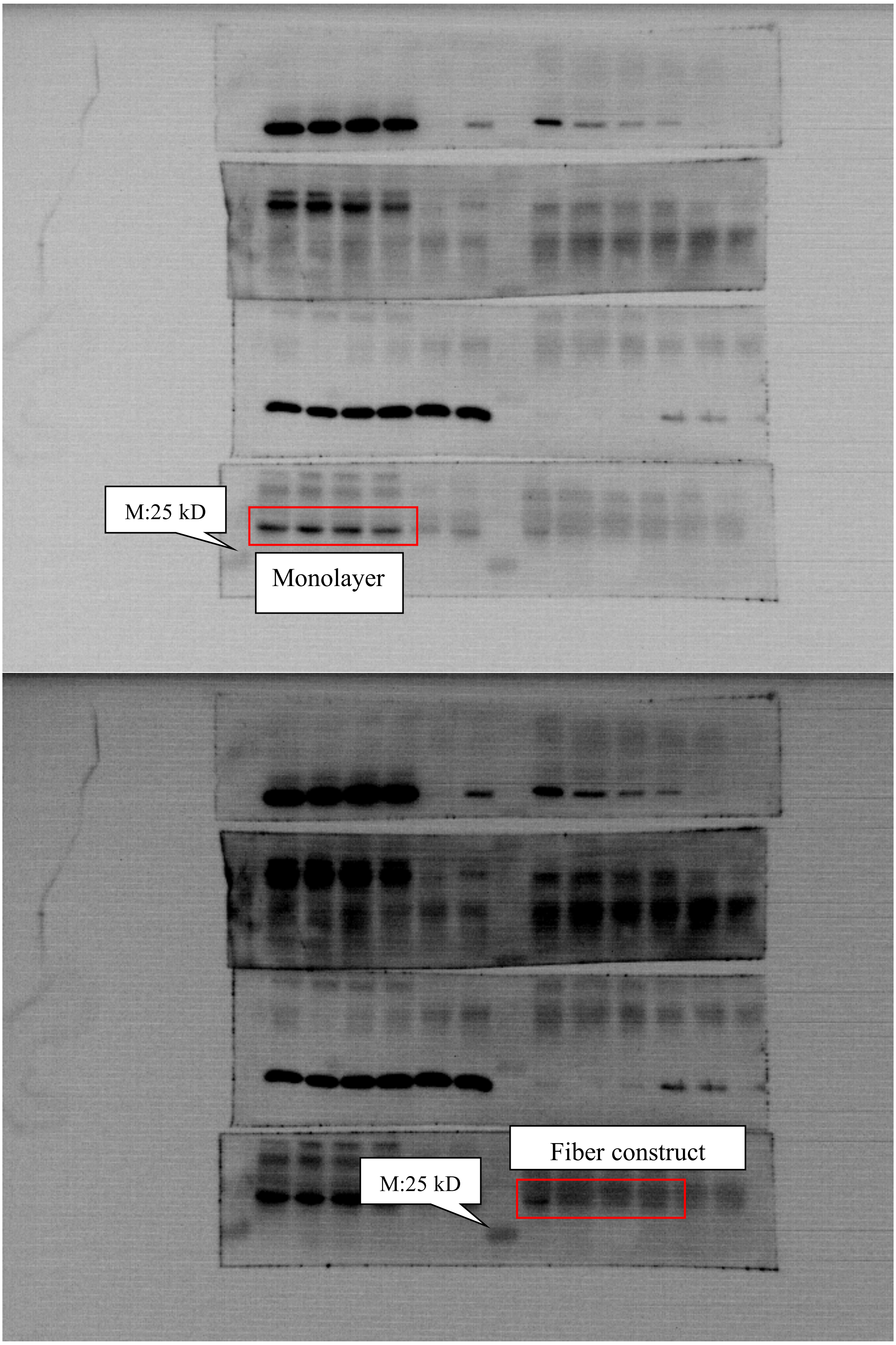

Figure 1.2 Acrylamide-Bcl-2

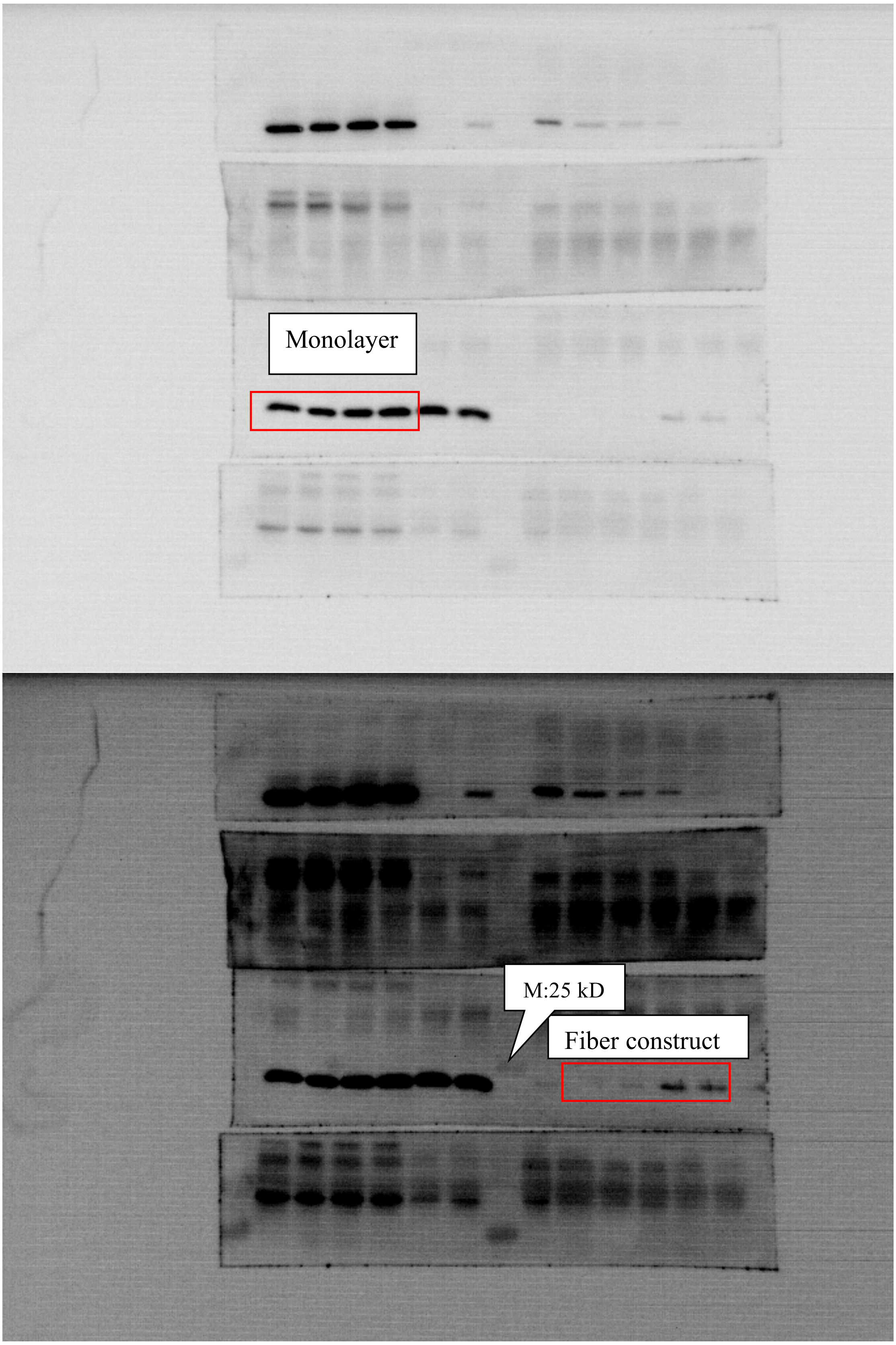

Figure 1.3 Acrylamide-bax

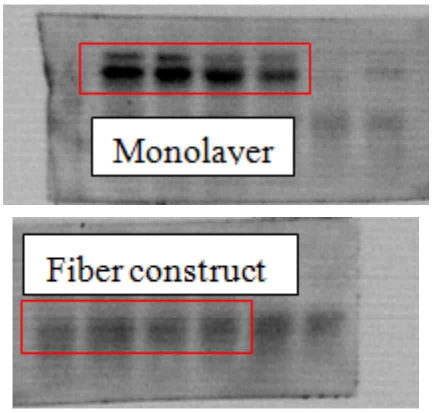

Figure 1.4 Acrylamide-Caspase3

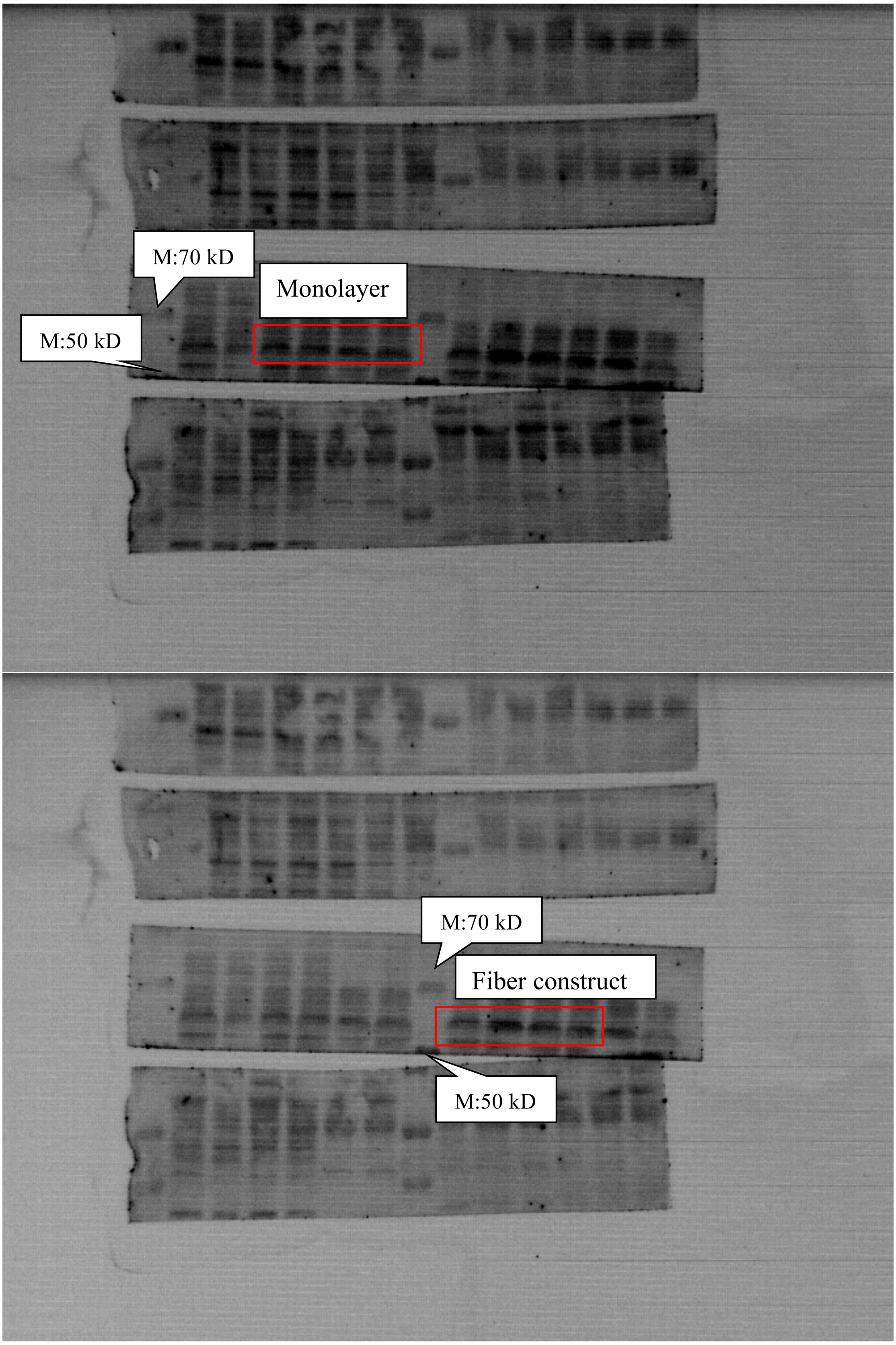

Figure 1.5 Acrylamide-p53

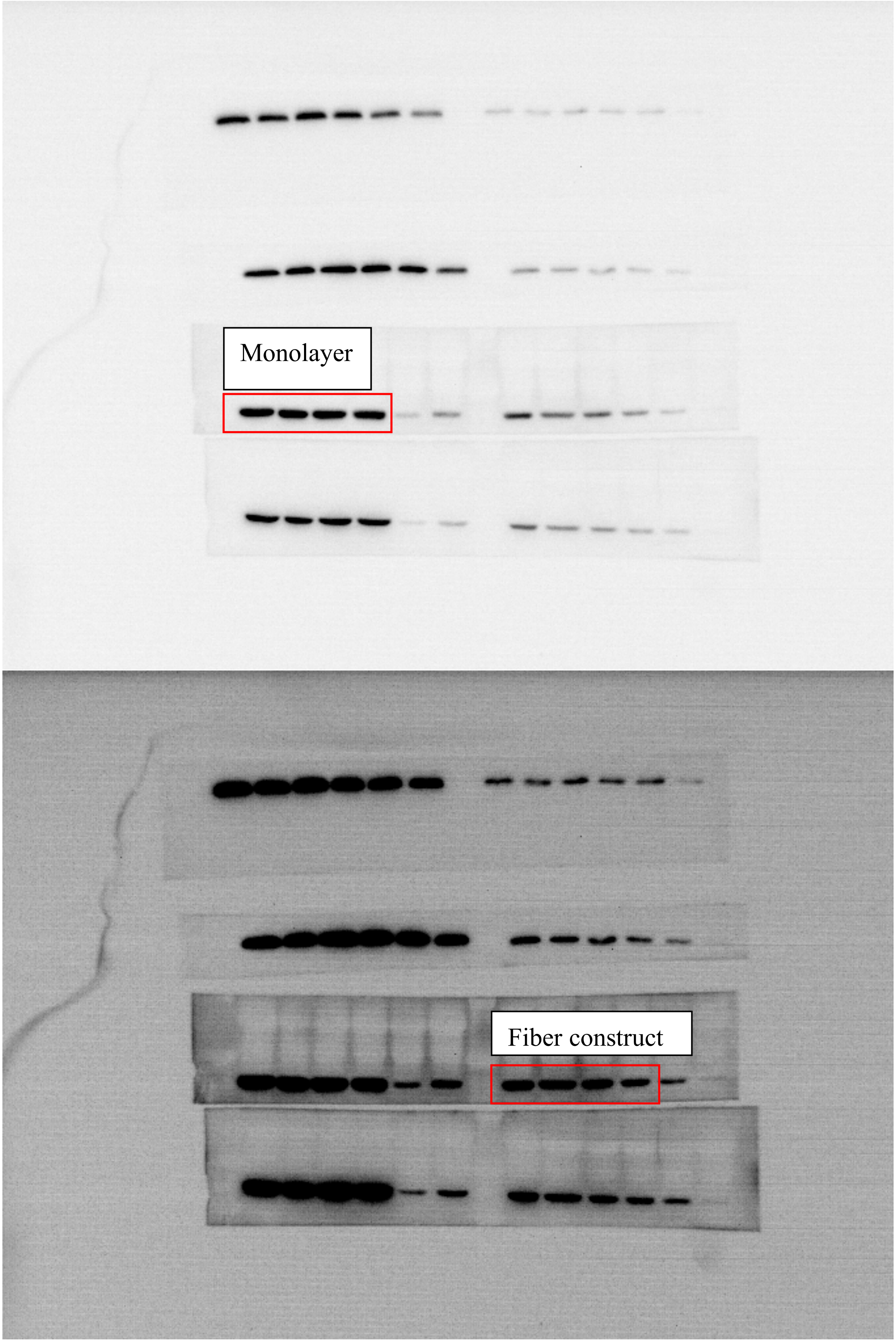

Figure 1.6 Acrylamide-β-actin

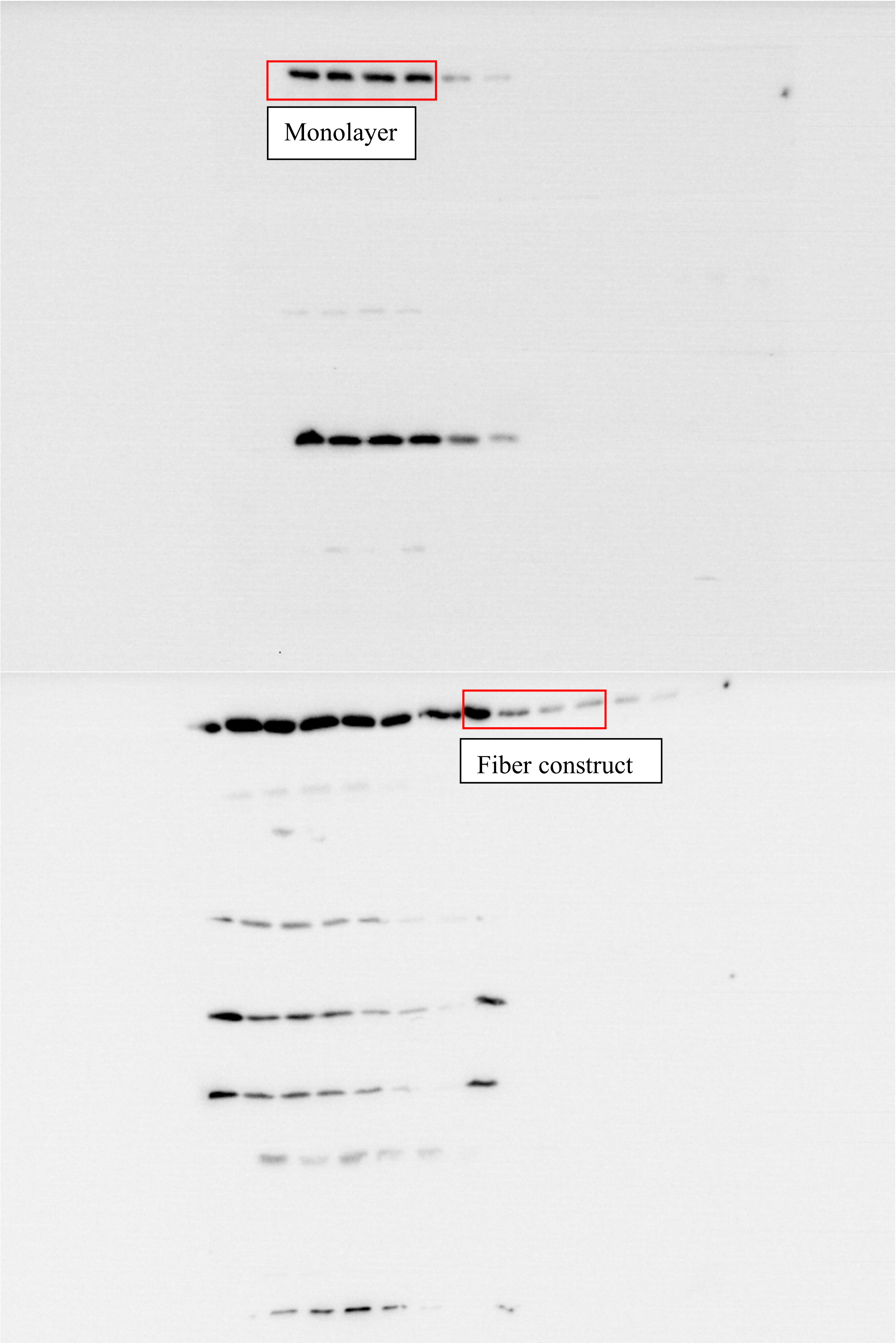

Figure 2.1 Sodium Nitrite-PCNA

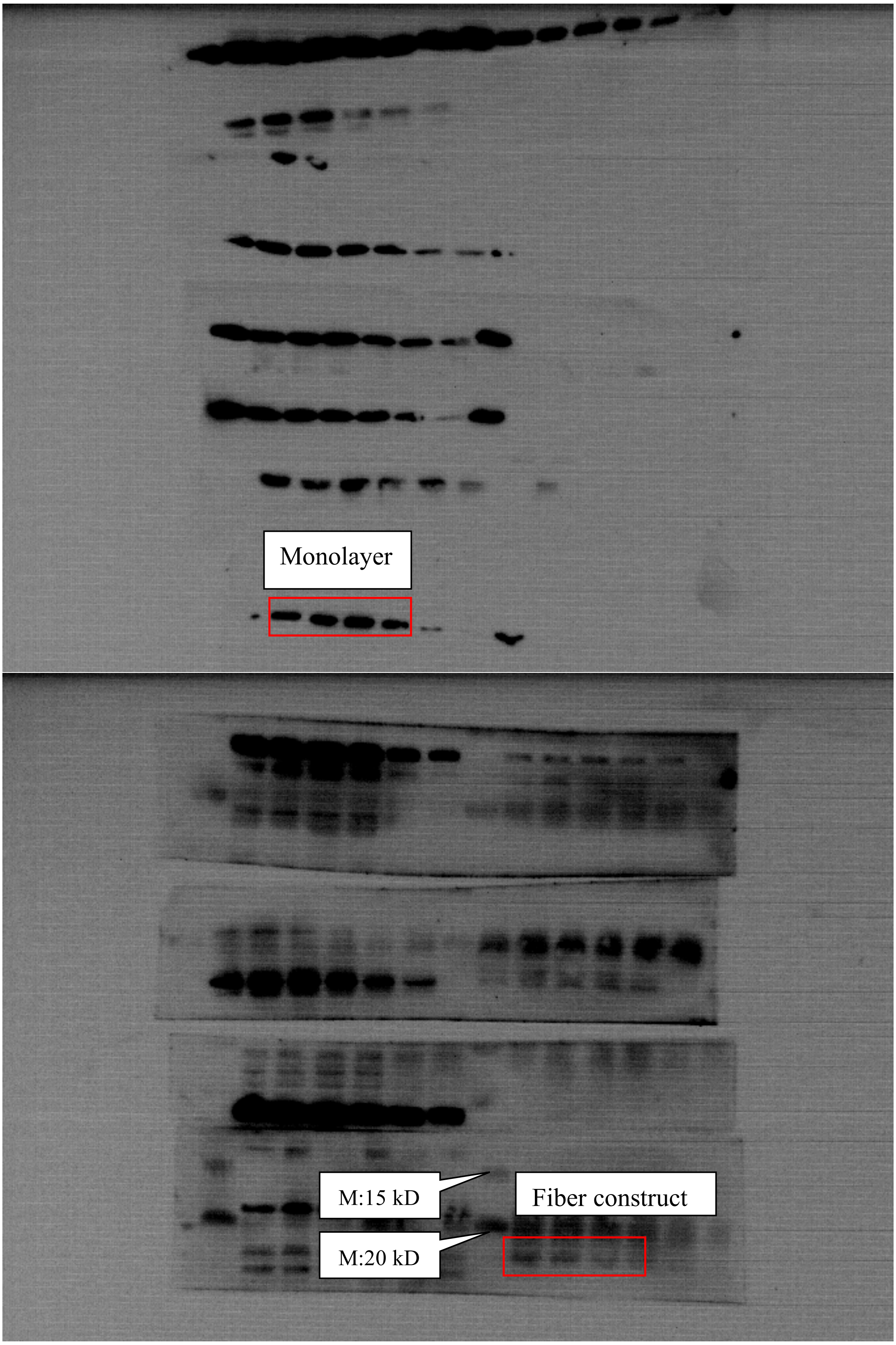

Figure2.2 Sodium Nitrite-Bcl-2

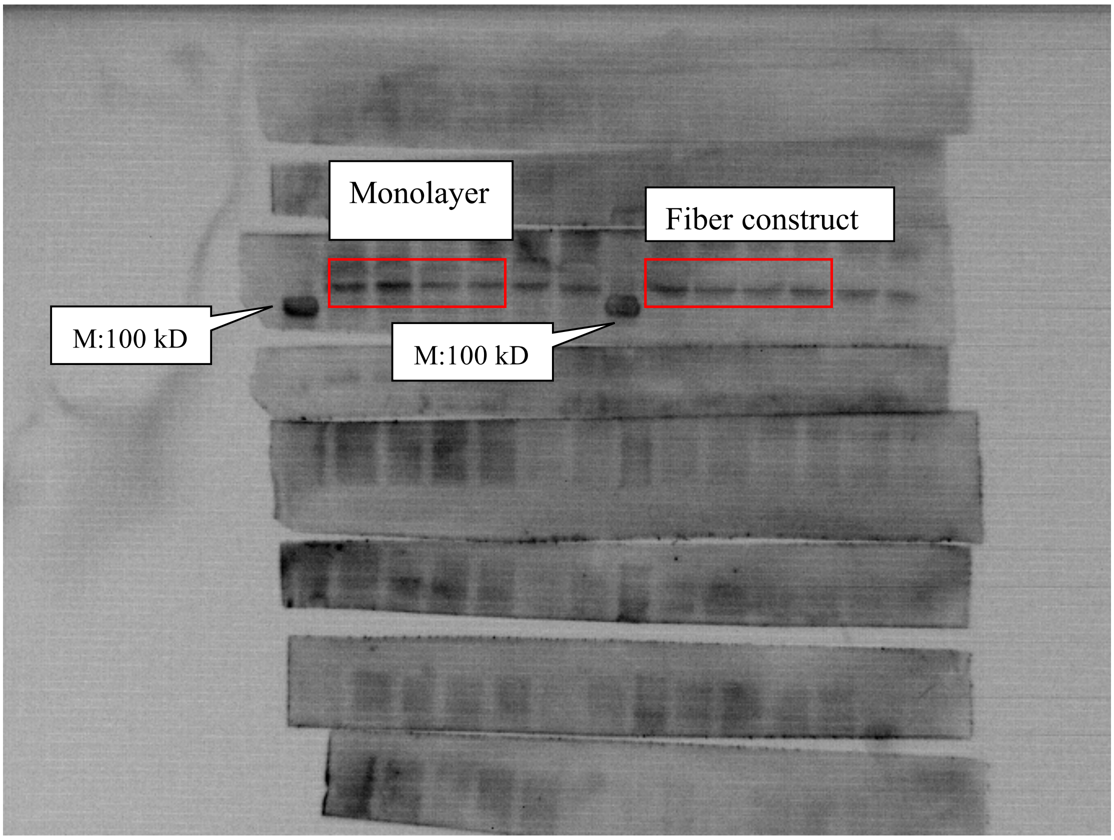

Figure 2.3 Sodium Nitrite-SIRT1

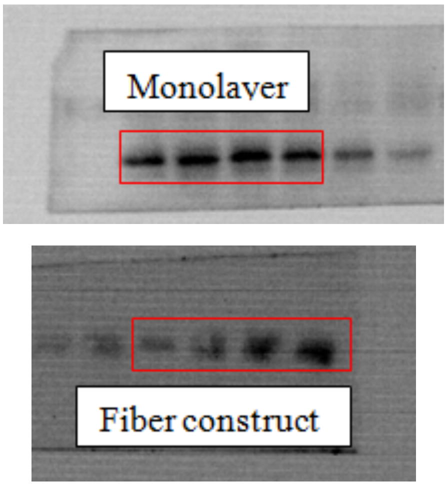

Figure 2.4 Sodium Nitrite-Caspase3

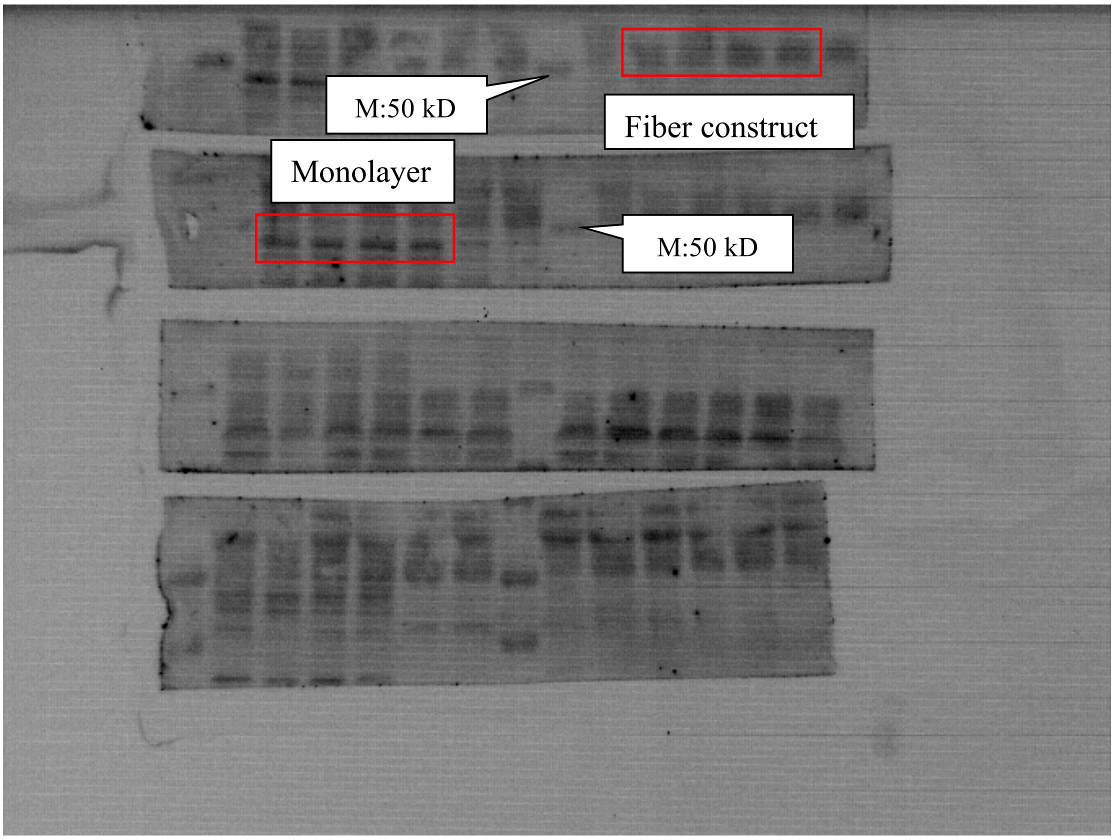

Figure 2.5 Sodium Nitrite-p53

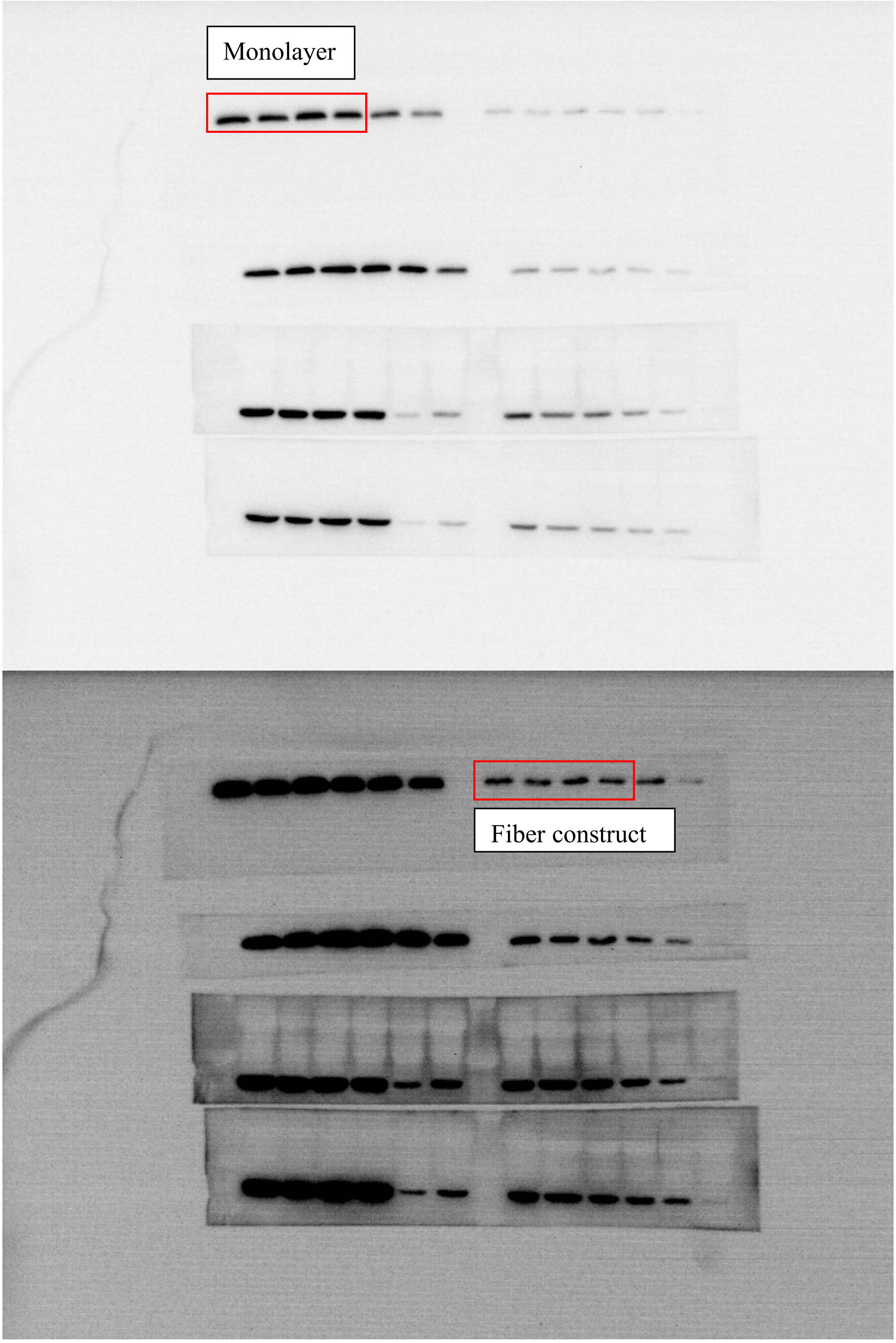

Figure 2.6 Sodium Nitrite-β-actin
